# Children’s Weight Gain and Cardiovascular Fitness Loss over the Summer

**DOI:** 10.3390/ijerph15122770

**Published:** 2018-12-07

**Authors:** Timothy A. Brusseau, Ryan D. Burns

**Affiliations:** Department of Health, Kinesiology, and Recreation, University of Utah, Salt Lake City, UT 84112, USA; ryan.d.burns@utah.edu

**Keywords:** health-related fitness, physical education, physical activity, summer breaks

## Abstract

The purpose of this study was to examine the impact of summer breaks on the body composition and cardiovascular fitness of elementary school children who participated in a multi-year school-based physical activity intervention. Participants were 404 children who had their height and weight measured and completed the Progressive Aerobic Cardiovascular Endurance Run (PACER) during physical education classes at the beginning and end of the school year for three consecutive years. To examine the effects of time on health-related fitness data, general linear mixed effects models were employed. The results indicate that there was a trend toward an increase in body mass index (BMI) after the summer of 2015 (*p* = 0.958), and a significant increase in BMI after the summer of 2016 compared to time point 1 (*p* < 0.001). For PACER laps, there were trends toward decreases in PACER laps after the summers of 2015 (*p* = 0.515) and 2016 (*p* = 0.073). Summer breaks tended to attenuate the BMI and PACER lap improvements that were observed during the intervention. While school-based physical activity programming has had some successes in improving health-related fitness markers, the loss of these improvements over the summer is of concern to both practitioners and researchers. It is clear that additional efforts are needed to limit obesogenic behaviors during the summer months.

## 1. Introduction

The benefits of physical activity and health-related fitness for children are well documented [1]. Unfortunately, children are falling short of the recommended levels of physical activity both habitually [2] and at school [3], and are often not meeting age and sex-specific criterion-referenced standards for health-related fitness [4]. Additionally, physical activity and health-related fitness either track or predict physical activity in adulthood [5,6].

To combat low levels of physical activity and health-related fitness, school-based interventions have been identified as ideal ways to change behavior [7]. Specifically, The Society for Health and Physical Educators of America (SHAPE) and the Centers for Disease Control and Prevention [8] recommend Comprehensive School Physical Activity Programs (CSPAP). CSPAPs are multi-component opportunities for children to be more active at school, which include physical education, recess, classroom-based physical activity, before and after school opportunities, staff involvement, as well as family and community engagement [8]. Studies have begun to explore the impact of CSPAP on a variety of outcomes showing positive change in both physical activity and health related fitness [9,10].

A recent concern with school-based physical activity interventions (e.g., CSPAPs) is the inability to continue these programs over the summer, which may negate health-related fitness improvements made during the academic year [11]. Numerous studies have highlighted increases in weight gain and fitness loss over the summer compared to the school year [12,13,14]. Schools often provide regular physical activity opportunities (e.g., physical education and recess), classroom teachers control screen time, parents often enforce bed and wake times in preparation for school, and schools often provide both breakfast and lunch programs that regulate diet [14]. An example of the impact of not having regular structure may be the changes seen when comparing physical activity on weekdays and weekend days. Studies continuously show children being more active during the week, and have attributed the large discrepancies to the elimination of daily and structured activity opportunities during school [15]. To the authors’ knowledge, no study to date has examined the trends in health-related fitness, specifically body weight status and cardiovascular fitness, across a multi-year CSPAP with the intent to examine changes in body mass index (BMI) as a proxy for body composition and cardiovascular fitness loss over multiple summer breaks. With the understanding that numerous national organizations are recommending schools adopt CSPAP and with the concerns relative to summer weight gain and fitness loss, the purpose of this study was to examine the impact of summer breaks (lack of structured school days) on body weight status (as assessed by BMI) and cardiovascular fitness in elementary school children who participated in a multi-year school-based physical activity intervention.

## 2. Materials and Methods

### 2.1. Study Design

This study utilized an observational repeated measures design. A repeated measure design was used to examine changes in health-related fitness over three consecutive years (six time points) with measures collected in the fall and spring for three consecutive school years. The first time point was the fall of 2014 and the last time point being the Spring of 2017.

### 2.2. Participants and Setting

Participants were children (*n* = 404; 203 girls, 201 boys) who were in grades one to four at time point 1 from three low-income elementary schools in a capital city in the southwestern United States who were participating in a school-based CSPAP intervention that had a primary focus on improving physical education, adding semi-structured physical activities to recess, and infusing classroom activity breaks and active academics (academic lessons done while being physically active) into their classrooms. Participants were 80% ethnic minority, and 88% received free or reduced lunch. Parental permission and student assent were ascertained prior to the start of the study. The University Institutional Review Board approved all of the procedures and methodology in the fall of 2014 (IRB_00078226).

### 2.3. Health-Related Fitness Measures

Body mass index (BMI) was calculated by taking a student’s weight in kilograms divided by the square or his or her height in meters (kg/m^2^). Height was measured to the nearest 0.01 m using a portable stadiometer (Seca 213, Hanover, MD, USA), and weight was measured to the nearest 0.1 kg using a portable medical scale (BD-590, Tokyo, Japan). Height and weight were measured without shoes in a semi-private area during physical education classes. Absolute BMI scores were used to make the results more interpretable, and because of minimal physical development over between time points.

Cardiovascular fitness was measured using the 20-m Progressive Aerobic Cardiovascular Endurance Run (PACER) [16], which was administered during each student’s physical education class. The PACER is a valid and reliable test for measuring cardiovascular fitness in children [16]. The PACER was conducted on a marked gymnasium floor with background music provided by a compact disk. Each student was instructed to run from one floor marker to another floor marker across a 20-m distance within an allotted time frame. The final score was recorded in laps. 

### 2.4. Procedures

All of the data were collected within the first four weeks and last four weeks of the school year. PACER tests were completed during physical education in sex-specific groups following established procedures [16]. The PACER test was concluded when the students were unable to reach the markers two times during the test. While one group of students completed the PACER, the other students had their height and weight measured in a semi-private area of the gym. These procedures were repeated six times, in the fall and spring for three consecutive academic years. 

### 2.5. Statistical Analysis

Health-related fitness data were screened for outliers using box-plots and *z*-scores and checked for Gaussian (normal) distributions using k-density plots, which are plots similar to histograms. Forty observations were deleted (9.9% of total sample) because of extreme BMI *z*-scores above or below 3.0 *z*. Data were approximately Gaussian distributed for both BMI and PACER laps after this data-cleaning procedure. BMI and PACER lap differences between sexes and among grade levels at time point 1 were examined with a two (gender) by four (grade level) factorial analysis of variance (ANOVA) tests, and one two by four ANOVA for BMI and PACER each, respectively. Bonferroni post hoc tests were employed if there was a statistically significant main effect for grade level. Effect sizes were examined using Cohen’s *d*, with *d* < 0.20 representing a small effect, *d* ≈ 0.5 representing a medium effect, and *d* > 0.80 representing a large effect [17]. Cohen’s *d* was calculated by dividing the mean differences by pooled standard deviation [17].

To examine the effects of time on health-related fitness data, general linear mixed effects models were employed. The parameter estimates of interest were the time main effect and the sex × time interaction. Particular attention was paid to examining the parameter estimates from time coefficients, which were treated as a categorical predictor. Sex was analyzed as a predictor on the categorical measurement scale. The referent for the time variable was fall 2014 (time point 1), and the referent for sex was males. Separate models were run for the BMI outcome and the PACER lap outcome. Random intercepts were employed on the student level (time observations clustered within students). School was not used as a higher level within the model because of the small number of clusters (*N* = 3). All of the analyses had an initial alpha level of *p* ≤ 0.05, and were analyzed using STATA v.14.0 statistical software package (College Station, TX, USA).

## 3. Results

**Figure 1:**
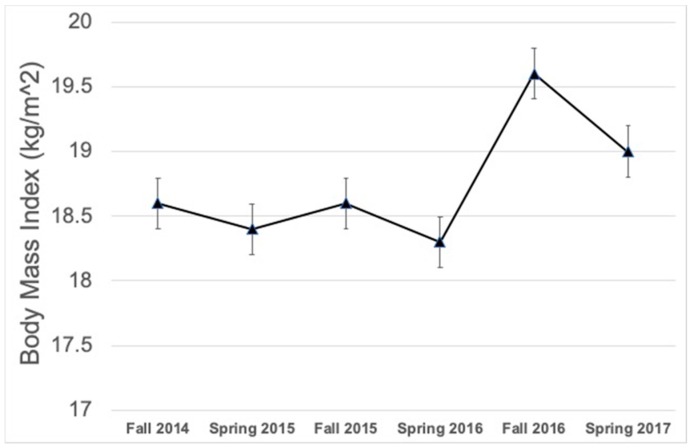
Mean BMI across time points for the total sample (means and standard errors; *n* = 404). *Note*: BMI stands for body mass index.

**Figure 2:**
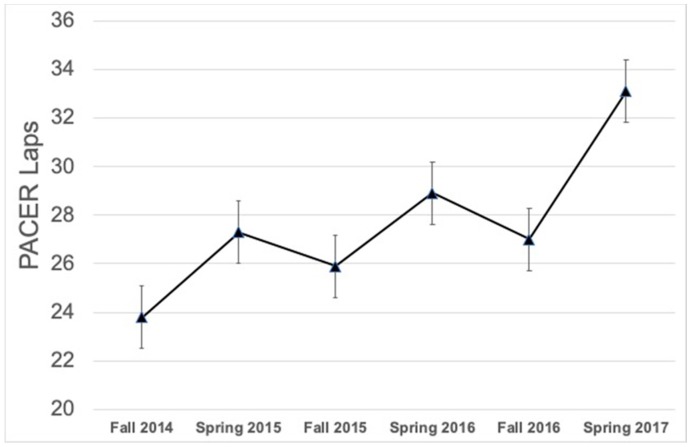
Mean PACER laps across time points for the total sample (means and standard errors; *n* = 404). *Note:* PACER stands for Progressive Aerobic Cardiovascular Endurance Run.

The descriptive statistics at the time point 1 are communicated in Table 1. Figure 1 and Figure 2 show the mean BMI and PACER laps across the six time points. There was a statistically significant grade level main effect yielding differences in BMI between grade three and grade one (mean difference = 2.0 kg/m^2^, *p* < 0.001, *d* = 0.52), between grade four and grade one (mean difference = 2.2 kg/m^2^, *p* < 0.001, *d* = 0.58), and between grade four and grade two (mean difference = 1.5 kg/m^2^, *p* < 0.001, *d* = 0.38). There were no differences between sexes regarding BMI. For PACER scores, there were significant differences between sexes (mean difference = 2.3 laps, *p* < 0.001, *d* = 0.15), with boys recording higher PACER laps than girls. There was also a statistically significant main effect for grade level on PACER laps, with differences in PACER laps between grade three and grade one (mean difference = 8.1 laps, *p* < 0.001, *d* = 0.62), and between grade four and grade one (mean difference = 7.6 laps, *p* < 0.001, *d* = 0.59). 

The results from the general linear mixed effects models for BMI and PACER time effects are communicated in Table 2. For BMI, there was a non-significant trend toward an increase in BMI after the summer of 2015, and a significant increase in BMI after the summer of 2016 compared to time point 1 (*p* < 0.001). Summer breaks attenuated the BMI improvements that were observed during the CSPAP intervention. For BMI, there was a non-significant trend toward an increase in BMI after the summer of 2015 (*b* = 0.1, *p* = 0.958), and a significant increase in BMI after the summer of 2016 compared to time point 1 (*b* = 1.3, *p* < 0.001). For PACER laps, there were trends toward decreases in PACER laps after the summers of 2015 (*b* = −0.5 laps, *p* = 0.515) and 2016 (*b* = −1.6 laps, *p* = 0.073); however the mean differences were not statistically significant compared to time point 1. Similar to BMI, summer breaks attenuated the PACER lap improvements that were observed during the intervention. Sex did not modify any of the observed time trends.

## 4. Discussion

The purpose of the current study was to examine the impact that multiple 12-week summer breaks have on the BMI and cardiovascular fitness of children participating in a school physical activity intervention over multiple years. The current study highlights a concerning trend that the improvements in health-related fitness made during the academic year are partially or completely negated during the summer months. 

Schools have been identified as an ideal locale for intervening with children to change physical activity behavior due to nearly all children regularly attending school, and there being the existing infrastructure to support physical activity programming [18]. To that end, a majority of interventions targeting obesity-related outcomes in children are taking place in schools [19]. As Weaver et al. [20] highlighted, school by itself may provide a natural intervention for preventing obesogenic behaviors, and summer is likely a larger contributor to fitness loss in children. While summer break has been linked to both weight gain and fitness loss [21,22], the concern may be even greater in low-income neighborhoods [23]. Children from low-income families typically have less opportunity and access to physical activity opportunities [24], which may highlight the trends in weight gain and fitness loss in the current study, as the children were primarily from low-income families. Brazendale et al., [14] through their proposed structured day hypothesis, illustrated how children on non-school days (weekends) have lower levels of physical activity, increased screen time, later bed and later wake times, and are more likely to consume unhealthy food and beverages. While school days likely protect children from the weight gain/fitness loss associated with these behaviors on weekends [14], up to 12 consecutive weeks of these behaviors are likely reflected in the summer fitness and weight changes from the current study as well as others.

Summer camps have been shown to help students exceed national physical activity recommendations [25]. Additionally, summer school programming has shown the ability to protect students from weight gain and fitness loss when compared to youth not participating in these programs [26]. A barrier to summer camp participation is the cost. The American Camp Association [27] noted that while financial assistance may be available, resident camp tuition ranges from $630 to over $2000 per week, and day camp tuition can range from $199 to over $800. Summer school programs are often available to children that need extra effort to meet their academic goals, but frequently have few spaces available, which reduces the number of students who participate. 

It is also important for schools, school personnel, and researchers who are implementing CSPAPs to consider ways to prepare children to be active during school breaks. While this study highlights the importance of programming over the summer, ideas for increasing physical activity on other non-school days (e.g., weekends, holidays, etc.) are also important. CSPAPs include family and community involvement as a key component for program success [8]. In fact, Cipriani, Richardson, and Roberts [28] have suggested that these are the most important components. More specifically, when family and community involvement are added to multi-component school-based interventions, children have greater increases in their physical activity [29]. The engagement of family members and the greater community would be key for summer programming that builds on school-based CSPAP. Cipriani et al. [28] suggested a number of strategies for increasing family and community involvement. They suggest the importance of increasing communication, and creating active events that involve family and the community. Physical education teachers and other school physical activity leaders have the opportunity to provide parents with information about and activities that children can participate in at home or with their families. Physical activity calendars, web resources, and quick facts can all be made available. Similar to information about physical activity, information about proper sleep and nutrition can be made available. Email and social media can be used to communicate and encourage improved physical activity, sleep, and nutrition behavior. School-based active events could be used during the school year to teach families and community members about a variety of anti-obesogenic behaviors, as well as provide opportunities for these groups to participate in health activities together. The physical education teacher can also monitor both physical activity and the health-related fitness of children during the academic year, and develop activity and exercise plans for the students to participate in over the summer or other school breaks. Ultimately, CSPAP and other school-based anti-obesogenic efforts need to account for the potential for fitness losses and weight gain over the summer months. 

A strength of the current study was the tracking of these outcomes over multiple summers using a reactively large sample within a longitudinal research design. Sex was also tested as a moderator variable. However, given these strengths, there are limitations that should be considered. The use of only three schools in one United States (US) city limits the ability for findings to be generalized to other groups of children. Similarly, while both the use of BMI and the PACER test are widely used in the pediatric literature, they are not direct measures of body composition and cardiovascular fitness. The use of physical education class time did not allow for measures to be completed in a fasted state, and each year, the classes may have been at different times. Lastly, this study did not track the physical activity opportunities of the children during the summer.

The results of this study manifest avenues for future research directions. Beets et al. [30] suggested that simply expanding, extending, or enhancing physical activity opportunities will lead to increases in physical activity in children. If this is not through formal school or camp programming, parents need to be aware of and encourage children to participate in the same healthy behaviors they do during school days. Unfortunately, there have been a limited number of home-based interventions examining these outcomes, and those that do exist have produced inconclusive results [31]. Additionally, school districts have increasingly been considering the use of a year-round school calendar [32]. This calendar eliminates the traditional 12-week summer break by spreading the 180-day school calendar over 12 months. Children typically have numerous two to three-week breaks throughout the year. While the impact of alternative school calendars on academic achievement has been researched with mixed findings [32,33], there have not been any studies examining the impact of the year-round school calendar on weight gain and fitness loss. A recent study by Weaver et al. [20] found that breaks as short as one week have a negative impact on sleep patterns, while a three-week break led to significant increases in sedentary time and decreases in physical activity. Future work is needed to explore the impact of year-round school calendars on health-related fitness.

## 5. Conclusions

While school-based physical activity programming has had some successes in improving health-related fitness markers, the loss of these improvements over the summer is of concern to both practitioners and researchers. It is clear that additional efforts are needed to limit obesogenic behaviors during the summer months. Researchers need to examine low-cost summer programming, including traditional summer school and camps, as well as home or family-based programming. Additionally, attention needs to be paid to year-round school calendars as a potential means of protecting children from unnecessary weight gain and fitness loss.

## Figures and Tables

**Table 1:** Means and (standard deviations) at time point 1 for body mass index (BMI) and PACER laps.

Sample	Body Mass Index (kg/m^2^)	PACER Laps
Total (*n* = 404)	18.6 (3.8)	23.9 (15.2)
Females (*n* = 203)	18.4 (3.2)	22.6 (14.7)
Males (*n* = 201)	18.7 (4.1)	25.3 * (16.4)
Grade 1 (*n* = 91)	17.9 (3.2)	18.3 (8.1)
Grade 2 (*n* = 102)	18.1 (3.5)	20.1 (11.2)
Grade 3 (*n* = 97)	**19.2 ^†^ (4.1)**	**25.1 ^†^ (14.5)**
Grade 4 (*n* = 114)	**19.5 ^††^ (4.4)**	**24.4 ^†^ (14.2)**

*Note*: PACER stands for Progressive Aerobic Cardiovascular Endurance Run; bold and * indicates statistical differences between sexes; bold and **^†^** indicates statistical differences compared to grade l; bold and **^††^** indicates statistical differences compared to grade 2 and grade 1, *p* ≤ 0.05.

**Table 2:** Time effect parameter estimates from the BMI and PACER general linear mixed effects models (*n* = 404).

Outcome	Time Level	*b*-Coefficient	95% Confidence Interval	*p*-Value
BMI (kg/m^2^)	Spring 2015	−0.2	−0.11, 0.7	0.111
	Fall 2015	0.1	−0.48, 0.47	0.958
	Spring 2016	−1.0	−1.9, −0.1	0.028
	Fall 2016	1.3	0.2, 2.4	<0.001
	Spring 2017	0.4	−0.52, 1.5	0.351
PACER (Laps)	Spring 2015	3.3	1.8, 4.8	<0.001
	Fall 2015	−0.5	−1.9, 0.9	0.515
	Spring 2016	2.4	0.1, 4.6	0.040
	Fall 2016	−1.6	−3.3, 0.2	0.073
	Spring 2017	4.8	1.7, 7.9	0.002

*Note*: BMI stands for body mass index; PACER stands for Progressive Aerobic Cardiovascular Endurance Run; the referent for time was time point 1 (fall 2014); bold indicates statistical significance, *p* ≤ 0.05.
